# 1,4-Bis[2-(prop-1-en­yl)phen­oxy]butane

**DOI:** 10.1107/S1600536811018538

**Published:** 2011-05-20

**Authors:** Musa R. Bayramov, Abel M. Maharramov, Gunay M. Mehdiyeva, Shahnaz B. Hoseinzadeh, Rizvan K. Askerov

**Affiliations:** a Baku State University, Z. Khalilov St 23, Baku AZ-1148, Azerbaijan

## Abstract

The mol­ecule of the title compound, C_22_H_26_O_2_, exhibits *C_i_* mol­ecular symmetry with a crystallographic inversion centre at the mid-point of the central C—C bond. A kink in the mol­ecule is defined by the torsion angle of 66.7 (2)° about this central bond of the alkyl bridge.

## Related literature

For general background to the use of copolymerization reactions, see: Crivello *et al.* (1994 ▶); Roshupkin & Kurmaz (2004 ▶); Askadsky (1998 ▶).
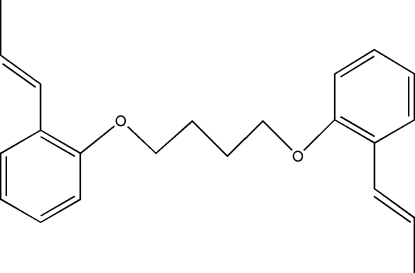


## Experimental

### 

#### Crystal data

C_22_H_26_O_2_
*M* *_r_* = 322.43
Orthorhombic, 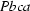

*a* = 5.4501 (10) Å
*b* = 15.825 (3) Å
*c* = 21.889 (4) Å
*V* = 1887.9 (6) Å^3^
*Z* = 4
Mo *K*α radiation
μ = 0.07 mm^−1^
*T* = 296 K
0.30 × 0.20 × 0.20 mm

#### Data collection

Bruker SMART APEXII CCD diffractometer
Absorption correction: multi-scan (*SADABS*; Bruker, 2001 ▶) *T*_min_ = 0.979, *T*_max_ = 0.986
20262 measured reflections
2404 independent reflections
1427 reflections with *I* > 2σ(*I*)
*R*_int_ = 0.040

#### Refinement

*R*[*F* ^2^ > 2σ(*F* ^2^)] = 0.042
*wR*(*F* ^2^) = 0.151
*S* = 1.01
2404 reflections
110 parameters
H-atom parameters constrained
Δρ_max_ = 0.15 e Å^−3^
Δρ_min_ = −0.11 e Å^−3^

### 

Data collection: *APEX2* (Bruker, 2005 ▶); cell refinement: *SAINT-Plus* (Bruker, 2001 ▶); data reduction: *SAINT-Plus*; program(s) used to solve structure: *SHELXTL* (Sheldrick, 2008 ▶); program(s) used to refine structure: *SHELXTL*; molecular graphics: *SHELXTL*; software used to prepare material for publication: *SHELXTL*.

## Supplementary Material

Crystal structure: contains datablocks global, I. DOI: 10.1107/S1600536811018538/kp2326sup1.cif

Structure factors: contains datablocks I. DOI: 10.1107/S1600536811018538/kp2326Isup2.hkl

Supplementary material file. DOI: 10.1107/S1600536811018538/kp2326Isup3.cml

Additional supplementary materials:  crystallographic information; 3D view; checkCIF report

## Figures and Tables

**Fig. 1.**
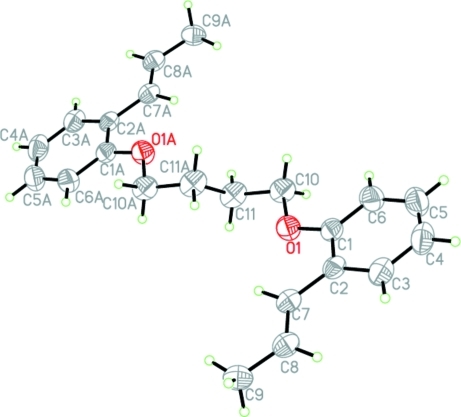
The molecular structure of the title compound with the atomic numbering scheme. Displacement ellipsoids were drawn at the 30% probability level.

**Fig. 2.**
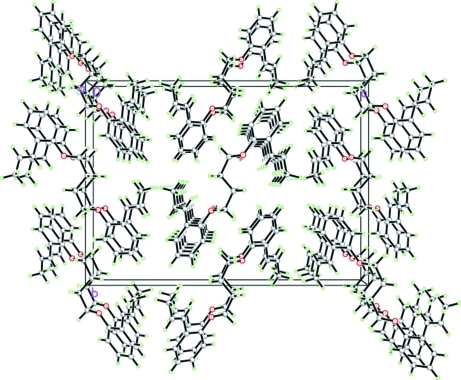
Stacking of chains in the crystal packing.

## supplementary crystallographic information

### Comment

For giving the important operationalisation to cross-linked polymers reactions of copolymerisation of various monomers, multifunctional comonomers [1] are used. By applying this method, the hi-tech processes for the preparation of polymeric sorbents, photorezisting materials for microelectronics, the composites for the laser technics of a special purposes, fixation of metalcomplex of catalysts, *etc* were obtained (Crivello *et al.* 1994). In practice, for obtaining polymers of demanded functional properties, polymerical transformations are carried out. However, it is necessary to notice, that obtaining such cross-linked copolymers have some difficulties connected with high reactivity of cross-linking comonomers (for example, divinylbenzene), which is reflected in heterogeneity of their structure and other important physical and chemical properties. Therefore, to prepare multifunctional monomers, on the basis of alkenylphenols with two double bonds, is rather important. The  molecule of the title compound, C_22_H_26_O_2_ (I), is generated by a crystallographic inversion centre at the midpoint of the central C—C bond. A fold of the molecule is due to the twist in the central butylene bridge [O1—C10—C11—C11A torsion angle of 66.7 (2)°] (Fig. 1). Crystal packing is dominated by van der Waals interactions (Fig. 2).

### Experimental

2-Propenylphenol (0.015 mol, 2 g) and KOH (0.015 mol, 0.84 g) were dissolved in 5 mL 2-propanol, then to this solution 1,4-dibromebutane (0.0043 mol, 0.93 g) was added. This mixture was stirred at 353 K within 30 min. The desired compounds (with yield of 4.7 g, 98.1%) was filtered and washed with acetone and recrystallised to obtain colourless crystals. Tmp = 353 K. The structure of the reported compound - 1,4-bis{2(1-propenyl)phenoxy}butane, also was proved by NMR-spectroscopy. FT-NMR (acetone-d6,, p.p.m.), 1H: 1.91 d (6*H*,CH3); 2.03 t (4*H*,CH2); 4.1 t (4*H*, OCH2); 6.15 m (2*H*, CH=); 6.65–7.1 m (8*H*, 2Ar); 7.32 d (2*H*,CH=). 13 C: 18.9; 26.3; 67.6; 112.8; 121.7; 124.9; 126.1; 127.2; 127.3; 127.5; 156.4.

### Refinement

The hydrogen atoms were placed in calculated positions and refined in the riding mode with fixed isotropic displacement parameters [*U*_iso_(H) = 1.2*U*_eq_(C)].

### Crystal data

**Table:**

C_22_H_26_O_2_	*F*(000) = 696
*M**_r_* = 322.43	*D*_x_ = 1.134 Mg m^−^^3^
Orthorhombic, *P**b**c**a*	Mo *K*α radiation, λ = 0.71073 Å
Hall symbol: -P 2ac 2ab	Cell parameters from 4034 reflections
*a* = 5.4501 (10) Å	θ = 2.6–23.1°
*b* = 15.825 (3) Å	µ = 0.07 mm^−^^1^
*c* = 21.889 (4) Å	*T* = 296 K
*V* = 1887.9 (6) Å^3^	Prism, colourless
*Z* = 4	0.30 × 0.20 × 0.20 mm

### Data collection

**Table:**

Bruker SMART APEXII CCD diffractometer	2404 independent reflections
Radiation source: fine-focus sealed tube	1427 reflections with *I* > 2σ(*I*)
graphite	*R*_int_ = 0.040
φ and ω scans	θ_max_ = 28.6°, θ_min_ = 1.9°
Absorption correction: multi-scan (*SADABS*; Bruker, 2001)	*h* = −7→7
*T*_min_ = 0.979, *T*_max_ = 0.986	*k* = −21→21
20262 measured reflections	*l* = −29→29

### Refinement

**Table:**

Refinement on *F*^2^	Primary atom site location: structure-invariant direct methods
Least-squares matrix: full	Secondary atom site location: difference Fourier map
*R*[*F*^2^ > 2σ(*F*^2^)] = 0.042	Hydrogen site location: inferred from neighbouring sites
*wR*(*F*^2^) = 0.151	H-atom parameters constrained
*S* = 1.01	*w* = 1/[σ^2^(*F*_o_^2^) + (0.071*P*)^2^ + 0.2*P*] where *P* = (*F*_o_^2^ + 2*F*_c_^2^)/3
2404 reflections	(Δ/σ)_max_ < 0.001
110 parameters	Δρ_max_ = 0.15 e Å^−^^3^
0 restraints	Δρ_min_ = −0.11 e Å^−^^3^

### Special details

**Table:**

Geometry. All e.s.d.'s (except the e.s.d. in the dihedral angle between two l.s. planes) are estimated using the full covariance matrix. The cell e.s.d.'s are taken into account individually in the estimation of e.s.d.'s in distances, angles and torsion angles; correlations between e.s.d.'s in cell parameters are only used when they are defined by crystal symmetry. An approximate (isotropic) treatment of cell e.s.d.'s is used for estimating e.s.d.'s involving l.s. planes.
Refinement. Refinement of *F*^2^ against ALL reflections. The weighted *R*-factor *wR* and goodness of fit *S* are based on *F*^2^, conventional *R*-factors *R* are based on *F*, with *F* set to zero for negative *F*^2^. The threshold expression of *F*^2^ > σ(*F*^2^) is used only for calculating *R*-factors(gt) *etc*. and is not relevant to the choice of reflections for refinement. *R*-factors based on *F*^2^ are statistically about twice as large as those based on *F*, and *R*- factors based on ALL data will be even larger.

### Fractional atomic coordinates and isotropic or equivalent isotropic displacement parameters (Å^2^)

**Table:**

	*x*	*y*	*z*	*U*_iso_*/*U*_eq_	
O1	0.1929 (2)	0.12834 (6)	0.05285 (5)	0.0764 (3)	
C1	0.1981 (3)	0.19881 (9)	0.08887 (6)	0.0641 (4)	
C2	0.3717 (2)	0.19780 (9)	0.13598 (6)	0.0614 (4)	
C3	0.3831 (3)	0.26839 (11)	0.17357 (8)	0.0805 (5)	
H3A	0.4969	0.2696	0.2052	0.097*	
C4	0.2297 (4)	0.33659 (12)	0.16510 (10)	0.0964 (6)	
H4A	0.2408	0.3832	0.1909	0.116*	
C5	0.0609 (4)	0.33587 (12)	0.11879 (11)	0.0958 (6)	
H5A	−0.0436	0.3817	0.1134	0.115*	
C6	0.0455 (3)	0.26750 (11)	0.08014 (8)	0.0826 (5)	
H6A	−0.0673	0.2675	0.0483	0.099*	
C7	0.5292 (3)	0.12253 (10)	0.14476 (7)	0.0655 (4)	
H7A	0.4863	0.0747	0.1225	0.079*	
C8	0.7203 (3)	0.11548 (10)	0.17993 (7)	0.0729 (4)	
H8A	0.7671	0.1632	0.2018	0.088*	
C9	0.8691 (3)	0.03876 (12)	0.18816 (7)	0.0789 (5)	
H9A	0.8709	0.0234	0.2306	0.118*	
H9B	1.0338	0.0494	0.1746	0.118*	
H9C	0.7998	−0.0065	0.1646	0.118*	
C10	0.0059 (3)	0.12144 (11)	0.00726 (8)	0.0791 (5)	
H10A	0.0261	0.1653	−0.0233	0.095*	
H10B	−0.1549	0.1277	0.0257	0.095*	
C11	0.0297 (4)	0.03563 (11)	−0.02174 (7)	0.0827 (5)	
H11A	0.1962	0.0286	−0.0366	0.099*	
H11B	−0.0797	0.0325	−0.0566	0.099*	

### Atomic displacement parameters (Å^2^)

**Table:**

	*U*^11^	*U*^22^	*U*^33^	*U*^12^	*U*^13^	*U*^23^
O1	0.0798 (7)	0.0745 (7)	0.0750 (7)	0.0052 (5)	−0.0182 (5)	−0.0066 (5)
C1	0.0641 (8)	0.0616 (8)	0.0665 (8)	−0.0057 (6)	0.0061 (7)	0.0056 (6)
C2	0.0609 (8)	0.0622 (8)	0.0611 (8)	−0.0133 (6)	0.0091 (6)	0.0018 (6)
C3	0.0864 (11)	0.0742 (10)	0.0807 (10)	−0.0152 (8)	0.0078 (9)	−0.0079 (8)
C4	0.1190 (15)	0.0636 (10)	0.1066 (14)	−0.0128 (10)	0.0252 (13)	−0.0162 (10)
C5	0.1026 (13)	0.0663 (11)	0.1185 (16)	0.0098 (9)	0.0153 (13)	0.0074 (10)
C6	0.0802 (10)	0.0741 (11)	0.0936 (12)	0.0050 (8)	0.0000 (9)	0.0135 (9)
C7	0.0640 (8)	0.0719 (9)	0.0605 (8)	−0.0116 (7)	−0.0001 (6)	−0.0039 (7)
C8	0.0725 (9)	0.0806 (10)	0.0657 (9)	−0.0124 (8)	−0.0047 (7)	−0.0068 (7)
C9	0.0681 (9)	0.0978 (12)	0.0708 (9)	0.0015 (8)	−0.0044 (7)	0.0010 (8)
C10	0.0803 (10)	0.0902 (11)	0.0666 (9)	−0.0064 (8)	−0.0164 (8)	0.0109 (8)
C11	0.0945 (12)	0.0979 (12)	0.0558 (8)	−0.0157 (10)	−0.0108 (8)	0.0026 (7)

### Geometric parameters (Å, °)

**Table:**

O1—C1	1.3659 (17)	C7—C8	1.300 (2)
O1—C10	1.4305 (19)	C7—H7A	0.9300
C1—C6	1.382 (2)	C8—C9	1.471 (2)
C1—C2	1.399 (2)	C8—H8A	0.9300
C2—C3	1.389 (2)	C9—H9A	0.9600
C2—C7	1.481 (2)	C9—H9B	0.9600
C3—C4	1.378 (3)	C9—H9C	0.9600
C3—H3A	0.9300	C10—C11	1.505 (2)
C4—C5	1.369 (3)	C10—H10A	0.9700
C4—H4A	0.9300	C10—H10B	0.9700
C5—C6	1.376 (3)	C11—C11^i^	1.511 (3)
C5—H5A	0.9300	C11—H11A	0.9700
C6—H6A	0.9300	C11—H11B	0.9700
			
C1—O1—C10	118.67 (12)	C7—C8—C9	125.81 (15)
O1—C1—C6	123.36 (14)	C7—C8—H8A	117.1
O1—C1—C2	115.47 (12)	C9—C8—H8A	117.1
C6—C1—C2	121.17 (15)	C8—C9—H9A	109.5
C3—C2—C1	117.23 (14)	C8—C9—H9B	109.5
C3—C2—C7	122.96 (14)	H9A—C9—H9B	109.5
C1—C2—C7	119.78 (12)	C8—C9—H9C	109.5
C4—C3—C2	121.55 (18)	H9A—C9—H9C	109.5
C4—C3—H3A	119.2	H9B—C9—H9C	109.5
C2—C3—H3A	119.2	O1—C10—C11	107.57 (13)
C5—C4—C3	120.05 (17)	O1—C10—H10A	110.2
C5—C4—H4A	120.0	C11—C10—H10A	110.2
C3—C4—H4A	120.0	O1—C10—H10B	110.2
C4—C5—C6	120.20 (18)	C11—C10—H10B	110.2
C4—C5—H5A	119.9	H10A—C10—H10B	108.5
C6—C5—H5A	119.9	C10—C11—C11^i^	112.91 (17)
C5—C6—C1	119.78 (18)	C10—C11—H11A	109.0
C5—C6—H6A	120.1	C11^i^—C11—H11A	109.0
C1—C6—H6A	120.1	C10—C11—H11B	109.0
C8—C7—C2	127.62 (14)	C11^i^—C11—H11B	109.0
C8—C7—H7A	116.2	H11A—C11—H11B	107.8
C2—C7—H7A	116.2		
			
C10—O1—C1—C6	−5.9 (2)	C3—C4—C5—C6	−0.7 (3)
C10—O1—C1—C2	174.45 (13)	C4—C5—C6—C1	1.1 (3)
O1—C1—C2—C3	179.89 (13)	O1—C1—C6—C5	179.48 (15)
C6—C1—C2—C3	0.3 (2)	C2—C1—C6—C5	−0.9 (2)
O1—C1—C2—C7	−1.52 (18)	C3—C2—C7—C8	−12.1 (2)
C6—C1—C2—C7	178.86 (14)	C1—C2—C7—C8	169.40 (15)
C1—C2—C3—C4	0.2 (2)	C2—C7—C8—C9	178.78 (15)
C7—C2—C3—C4	−178.36 (15)	C1—O1—C10—C11	−175.35 (13)
C2—C3—C4—C5	0.0 (3)	O1—C10—C11—C11^i^	66.7 (2)

Symmetry codes: (i) −*x*, −*y*, −*z*.
